# Learning instance-specific counterfactual models for continuous treatments using hypernetworks

**DOI:** 10.3389/frai.2026.1819009

**Published:** 2026-05-08

**Authors:** Roger Pros, Jordi Vitrià

**Affiliations:** Departament de Matemàtica i Informàtica, Universitat de Barcelona, Barcelona, Spain

**Keywords:** causal inference, continuous treatment effect estimation, counterfactual outcomes, hypernetworks, representation learning

## Abstract

In recent years, non-linear machine learning techniques have attracted increasing attention for estimating treatment effects from observational data. While most existing methods focus on binary treatment scenarios, the estimation of treatment effects for continuous interventions remains a critical challenge in many real-world applications. In this work, we introduce a novel neural network-based approach for estimating continuous treatment effects by leveraging hypernetworks to model counterfactual outcomes across treatment levels. This approach extends the principles of binary treatment effects computation to the continuous domain, addressing the key challenge of treatment relevance. By generating weights for a fixed network that predicts potential outcomes, our architecture ensures that the treatment variable retains its causal significance while maintaining the flexibility of deep learning models. Through extensive experiments on synthetic and semi-synthetic datasets, we demonstrate that our approach outperforms existing methods in terms of precision. The results highlight the advantages of explicitly modeling the relationship between treatment levels and outcomes, particularly in settings where traditional methods struggle with high-dimensional confounders or non-linear treatment-response dynamics.

## 1 Introduction

Estimating treatment effects is a fundamental objective across a wide range of domains, including clinical trials, economic policy evaluation, educational assessments, social science research, and artificial intelligence. In the latter, a key challenge in causal machine learning is to quantify the impact of specific interventions on outcomes and to predict counterfactual outcomes under alternative treatment conditions (Kaddour et al., 2022).

A central difficulty in this setting is the inherent uncertainty surrounding unobserved counterfactuals, commonly known as the “Fundamental Problem of Causal Inference” (Holland, 1986). For any given unit, only the outcome corresponding to the administered treatment is observable, while outcomes under alternative interventions remain unknown. This limitation often leads to biased estimates when conventional data-driven methods are applied without accounting for this missing data structure.

Recent advances have seen growing interest in leveraging high-capacity machine learning models to address these limitations and enhance causal effect estimation (Cavique, 2024). These methods seek to integrate the predictive capabilities of modern learning algorithms with the structural assumptions necessary for valid causal inference. Notable approaches include the use of tree-based models, kernel methods, and Gaussian processes, often in conjunction with techniques such as propensity score adjustment, matching, or covariate balancing to mitigate confounding. A particularly active and promising direction within this literature involves neural network-based models (Shalit et al., 2017; Shi et al., 2019; Tesei et al., 2023), which constitute the primary focus of the present study.

To overcome the challenges present in causal inference, recent research has focused on incorporating causal reasoning into representation learning frameworks (Schölkopf et al., 2021). These approaches aim to learn data representations that preserve the underlying causal structure. By incorporating assumptions such as unconfoundedness or leveraging balancing techniques, these models attempt to ensure that comparisons across treatment groups remain valid even in the presence of complex, high-dimensional data.

**Figure 1:**
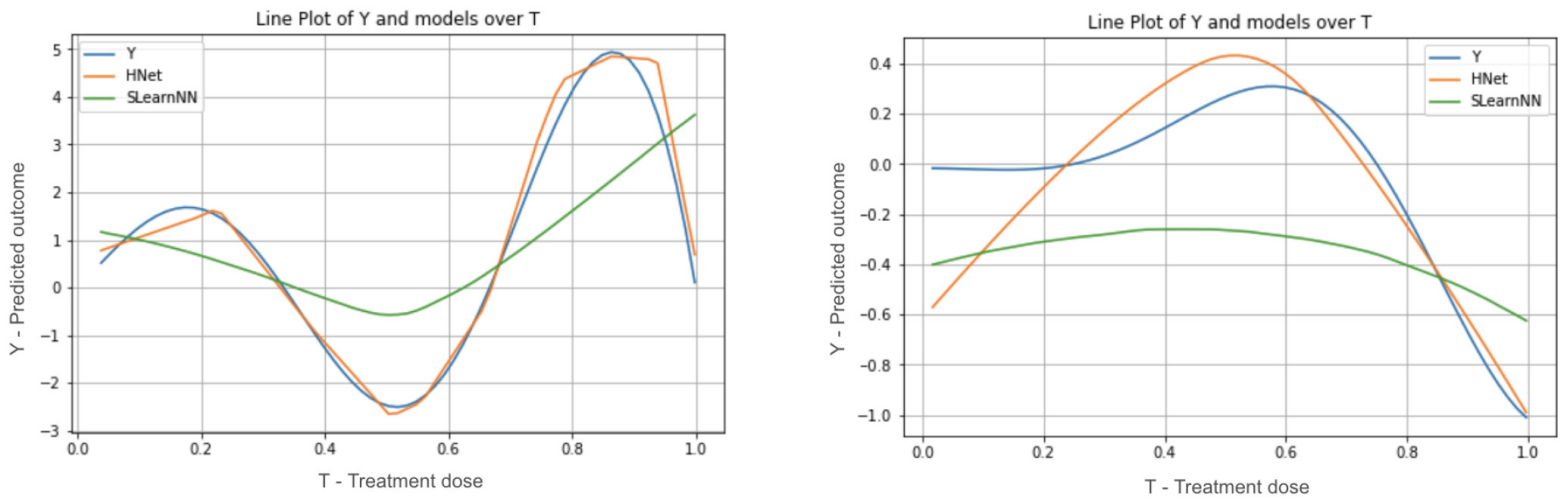
Illustrative comparison between a naive neural network-based S-learner (SLearnNN) and our proposed method (HNet). The *x*-axis (T) represents the continuous treatment variable, and the *y*-axis shows the outcome values. These examples are generated using synthetic data from Nie et al. (2021) to demonstrate model behavior. The naive model, which lacks treatment relevance bias, exhibits reduced sensitivity to the treatment variable, resulting in a poorer estimation of the true response curve compared to HNet.

A key requirement for the successful application of machine learning to causal inference is the explicit modeling of treatment variables independently from other covariates. Failing to do so risks obscuring treatment information within the latent representation, particularly in high-dimensional contexts, which can severely impair counterfactual estimation accuracy (Künzel et al., 2019), illustrated in Figure 1.

While substantial progress has been made in the binary treatment setting, extending these techniques to continuous treatments remains a significant challenge. Unlike binary treatments, where the task involves comparing discrete alternatives (e.g., treatment vs. control), continuous treatments introduce a continuum of possible interventions. This makes counterfactual estimation more complex, as it requires modeling how outcomes change smoothly across the entire range of treatment intensities. Moreover, the uncountable set of potential outcomes complicates both model training and validation, as conventional metrics or techniques used for binary treatments do not easily generalize. For instance, estimating the effect of varying a drug dosage on patient health demands learning a nuanced, fine-grained relationship between dose and outcome, which is sensitive to both non-linearities and confounding. These complexities highlight the need for specialized methods that can effectively handle the intricacies of continuous treatment regimes.

In this work, we propose HNet, a novel method that builds on insights from prior architectures developed for binary or discrete treatment effect estimation—such as TARNet, Dragonnet, DRNet, and HydraNet (Shalit et al., 2017; Shi et al., 2019; Velasco et al., 2022; Schwab et al., 2020)—to estimate individualized treatment effects in the continuous treatment setting. Our approach is designed to retain the advantages of representation learning while addressing the unique challenges posed by continuous treatment regimes.

In Section 2, we define the problem of estimating individualized treatment effects in the continuous treatment setting and outline the key assumptions required for estimation. In Section 3, we review related work on causal inference and discuss existing machine learning approaches. In Section 4, we present the architecture and learning objectives of HNet in detail. In Section 5, we evaluate HNet on benchmark datasets and compare its performance to existing baselines. Finally, in Section 6, we discuss our findings, limitations, and potential directions for future work.

## 2 Materials and methods

### 2.1 Problem statement and assumptions

We consider a setting where we observe a finite number *N* of samples (*Y, T, X*), where *Y* is the target variable, *T* is the treatment variable—potentially continuous, i.e., ${\left\{t_{i}\in {\mathbb{R}}^{\tau }\right\}}_{i=1}^{N}$—and *X*, the covariates, are given as ${\left\{x_{i}\in {\mathbb{R}}^{p}\right\}}_{i=1}^{N}$. In this context, we assume there exists a function *f*(*X, T*) that generates outcomes *Y*, and our goal is to estimate the function that characterizes the conditional causal effect of *T* on *Y*, given *X*.

In the case of binary treatments, we are typically interested in estimating the difference in potential outcomes, or counterfactuals, under treatment and control, that is, the contrast between receiving the treatment (*T* = 1) and not receiving it (*T* = 0). On the other hand, for continuous treatments, the goal is usually to estimate counterfactual outcomes under alternative treatment levels. Given a treatment value *T*, we aim to model how the outcome would change under different treatment values by learning a counterfactual function θ_*X*_(*T*) for each *X*: θ_*X*_(*T*) = *E*_*X*_[*f*(*do*(*T*), *X*)∣*X*]^1^. These objectives can be summarized through the following general expression for the conditional effect:


$$\begin{array}{l} \Phi (X)=\frac{\Delta f(X,T|X)}{\Delta T}=\{\begin{array}{ll} E_{X}[f(do(T=1),X) & \text{ if }T\in \{0,1\}\\ -f(do(T=0),X)|X] & \;\\ f(T,X)-\theta_{X}\left(T^{\prime }\right) & \text{ if }T\in \mathbb{R}\\ \forall T^{\prime }\in \mathrm{supp}(T) & \; \end{array} \end{array}$$

This formulation highlights the distinction between the discrete and continuous cases: in the binary case, the effect is a simple difference in outcomes; in the continuous case, it requires modeling a full counterfactual trajectory over treatment values.

Causal inference is inherently difficult because the causal quantity of interest cannot be directly observed in the data. Consequently, certain assumptions are required to use statistical estimands to answer causal questions. In this work, we operate under the standard assumptions needed to ensure identifiability of the causal effect (Bica et al., 2020):

Assumption 1 (Positivity):


$$\begin{array}{l} \forall x\in X\text{such that}p\left(x\right)>0,\text{we have}0<p\left(t|x\right)<1\text{for each}t\in T. \end{array}$$

This assumption ensures that every treatment level has a non-zero probability of occurring for all covariate profiles with positive density.

Assumption 2 (Conditional Unconfoundedness / Backdoor Criterion): the covariates *X* form a sufficient adjustment set. More specifically, *X* satisfies the backdoor criterion relative to treatment *T* and outcome *Y* if: *X* blocks all backdoor paths from *T* to *Y*, and *X* does not contain any descendants of *T*.

Assumption 3 (Consistency): if the intervention *do*(*T* = *t*) is performed, the observed outcome *Y* is equivalent to the outcome that would have been observed under that specific treatment assignment.

Together, these assumptions justify the identification of the interventional expectation via covariate adjustment: (Pearl, 2009; Robins and Hernan, 2008):


$$\begin{array}{l} E_{X}\left[Y|do\left(T\right),X\right]=E_{X}\left[Y|T,X\right]. \end{array}$$

### 2.2 Individualized treatment effects estimation with continuous treatment

Individualized treatment effect (ITE) estimation focuses on quantifying the causal effect of a treatment *T* on an outcome *Y* for a specific individual characterized by covariates *X*. However, in observational settings, only one potential outcome is observed per individual, giving rise to what is known as the fundamental problem of causal inference. In practice, this is addressed by conditioning on the entire feature space *X* to estimate the Conditional Average Treatment Effect (CATE). Under assumptions 1 and 2, CATE can be expressed using statistical estimands based on the observed data distributions.

When the treatment variable *T* is binary, taking values in {0, 1}, CATE can be written as the difference in conditional expectations: τ(*x*) = *E*[*Y*∣*T* = 1, *X* = *x*]−*E*[*Y*∣*T* = 0, *X* = *x*]. At the population level, this leads to the average treatment effect (ATE), given by *E*_*X*_[*E*[*Y*∣*T* = 1, *X*]−*E*[*Y*∣*T* = 0, *X*]]. Although this formulation appears straightforward, accurate estimation of treatment effects poses several challenges. First, the treatment variable T plays a fundamentally different role from the covariates X in causal inference, necessitating specialized modeling strategies (Künzel et al., 2019). Second, the process by which treatment is assigned can result in imbalanced data distributions. This imbalance may cause models to perform better when predicting outcomes under one treatment condition than the other, thereby introducing bias (Shalit et al., 2017). Third, even models that explicitly account for the special role of *T* often struggle to utilize training data efficiently across treatment groups, leading to inefficiencies in estimation (Künzel et al., 2019).

To address these challenges, a range of methods have been proposed. Two of the most widely used approaches for estimating binary treatment effects are the S-Learner and the T-Learner (Robins, 1986; Künzel et al., 2019; Nyberg and Klami, 2024). The S-Learner trains a single predictive model using both the treatment indicator and the covariates as input features. This model is then used to predict potential outcomes under both treatment conditions. However, a known limitation of the S-Learner is its tendency to shrink treatment effect estimates toward zero, especially in high-dimensional feature spaces. In contrast, the T-Learner partitions the data by treatment assignment and fits a separate model for each group. This approach mitigates the risk of underestimating the treatment effect when the treatment signal is weak, but it may suffer from reduced data efficiency due to the split in training samples.

More recent advances incorporate neural networks and representation learning to improve estimation. Models such as TARNet and Dragonnet (Shalit et al., 2017; Shi et al., 2019) introduce architectural inductive biases that promote balanced representations of treated and control groups, enhancing the estimation of treatment effects.

#### 2.2.1 From binary to continuous treatment effect estimators

A particularly influential class of models for treatment effect estimation leverages neural networks. Owing to their highly flexible architectures, neural networks are well-suited for incorporating inductive biases that enhance causal inference. A wide range of approaches have been proposed for both binary and continuous treatments within this framework.

In the binary treatment setting, TARNet (Shalit et al., 2017) introduces a shared feature representation that is followed by two separate output heads—one for each treatment group. This design ensures that the model retains sensitivity to the treatment assignment variable T, rather than treating it as a standard covariate. Dragonnet (Shi et al., 2019) extends this architecture by adding a third output head dedicated to estimating the propensity score, defined as *g*(*X*) = *P*(*T* = 1∣*X*). This approach reduces the bias caused by having different modeling performances for each value of the treatment. More complex approaches have also been successful. For example Chauhan et al. (2024) propose a deep learning framework called HyperITE that employs “soft weight sharing” via hypernetworks to dynamically share information between treatment groups when estimating individualized treatment effects (ITEs). By generating the weights of potential outcome networks conditioned on treatment identity, this approach enables end-to-end inter-treatment parameter sharing—leading to improved estimation accuracy, especially on small observational datasets. This approach is extended to composite outcomes and treatments in Chauhan et al. (2025). In a complementary direction, Kiriakidou et al. (2024) propose enhancing estimation by incorporating the average outcomes of neighboring units in both treatment and control groups as informative inputs, which can be seamlessly combined with the aforementioned methods.

Moving beyond binary treatment effects, HydraNet (Velasco et al., 2022), designed for discrete multi-level treatments, creates a separate “outcome head” for each treatment level off a common representation backbone. This architecture enables simultaneous learning of all potential outcomes while sharing information across levels, reducing variance and improving generalization across treatment arms. DRNet (Schwab et al., 2020) extends dose-response estimation to discrete treatments with continuous dosage by partitioning the treatment space into multiple strata and assigning a dedicated network head to each interval. A shared base network learns global representations, while each stratum-specific head captures localized effects—with regularization methods like distribution matching and propensity dropout helping reduce treatment assignment bias.

#### 2.2.2 Neural networks as continuous treatment estimators

In settings where treatment is continuous rather than binary, the treatment effect is defined for every pair of treatment levels for each single individual. Under this scenario, we are often interested in estimating counterfactual outcomes under alternative treatment levels.

Estimating continuous treatment effects introduces additional complexities beyond those encountered in binary treatment effect estimation. In particular, the continuous nature of the treatment variable *T* renders many of the most effective strategies developed for binary causal inference inapplicable or ineffective.

To address these challenges, methods often adopt inductive biases similar to those used in binary settings. For instance, the T-Learner becomes infeasible in the continuous case, as the treatment space cannot be partitioned into discrete groups. In contrast, the S-Learner retains its applicability, modeling all potential outcomes within a unified framework, and thus remains a valid candidate for continuous treatment effect estimation.

Several neural network-based models have been proposed to tackle the challenges of continuous treatment effect estimation. SCIGAN (Bica et al., 2020) adapts Generative Adversarial Networks (GANs) to the causal inference context by learning to generate counterfactual outcomes under continuous treatments. It jointly models treatment assignment and potential outcomes, enabling flexible, data-driven estimation of individualized dose-response curves. VCNet (Nie et al., 2021) addresses continuous treatment effect estimation by modeling the dose-response function using a neural architecture based on B-spline basis expansions. This design allows the model to capture smooth variations in treatment effects while maintaining flexibility. Furthermore, VCNet separates covariate representation from treatment effect modeling, thereby enhancing both interpretability and estimation stability. GIKS (Nagalapatti et al., 2024) introduces a kernel-based approach that leverages implicit kernel summation within a neural architecture. It estimates potential outcomes as a weighted sum of observed outcomes, with the weights learned via a differentiable kernel mechanism conditioned on both covariates and treatment values. This enables flexible, non-parametric estimation of dose-response functions while ensuring computational efficiency, and can be combined with other models.

Other recent advancements focus on dynamic weighting and distributional robustness. For instance, TransTEE (Zhang et al., 2022) shares our motivation for instance-adaptive treatment modeling by employing Transformer-based attention mechanisms. Similarly, ADMIT (Wang et al., 2022) introduces a neural dose-response estimator that utilizes distribution-aware training to handle the complexities of continuous treatments. While these approaches address similar fundamental challenges, our work explores an alternative architectural route by directly generating the weights of a target outcome network, enforcing a strict separation between representation learning and treatment application.

**Figure 2:**
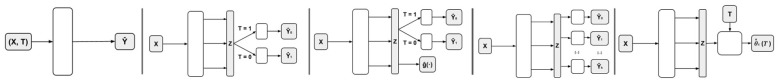
Evolution of neural architectures for treatment effect estimation. From left to right: SLearn (Künzel et al., 2019), TARNet (Shalit et al., 2017), Dragonnet (Shi et al., 2019), DRNet (Schwab et al., 2020), and our proposed model, HNet. Earlier models (e.g., S-Learner, TARNet) are designed for binary treatments and use shared representations with limited output heads. As the focus shifts to continuous treatments, architectures become more specialized—culminating in HNet, which dynamically generates a specific model for each individual to capture fine-grained treatment-response relationships.

A noticeable trend, illustrated in Figure 2, involves encoding treatment relevance biases in the representation layer of neural networks. The S-Learner does not explicitly model treatment-specific biases, as it produces a single output for all potential outcomes. TARNet introduces treatment relevance by employing two separate outcome heads—one for each treatment group—encouraging the model to learn treatment-specific patterns. Dragonnet extends this idea by adding a third output head to estimate treatment propensity, thereby addressing selection bias through targeted regularization. In the context of discrete (multi-level) treatments, HydraNet creates one outcome head per treatment level, enabling the model to learn separate responses for each treatment while sharing a common representation backbone.

Building on this progression, we propose a novel approach that, instead of allocating a distinct output layer per treatment level, utilizes hypernetwork principles to produce a fixed network that models θ_*X*_(*T*) for each individual observation. The following section provides a detailed description of this implementation strategy.

### 2.3 Method

In this section, we introduce a comprehensive methodology for constructing a model that leverages insights obtained from binary treatment effects computation to estimate continuous treatment effects. In particular, we introduce the treatment relevance condition while maintaining the benefits of other neural network estimators. We achieve this by using the concept of hypernetworks (Ha et al., 2016). A main network predicts a set of weights that are used to create a fixed network which approximates the counterfactual function θ_*X*_(*T*) for each single observation by estimating the outcome Ŷ. By integrating this principle into model design, we aim to improve the performance of the model in continuous treatment effects tasks.

#### 2.3.1 Architecture: hypernetwork

To operationalize this principle, we propose an inductive bias applied at the post-representation stage of a causal inference model. Most state-of-the-art approaches to estimating treatment effects begin by learning a representation of the covariates; we follow this general structure but introduce a key innovation in how potential outcomes are estimated from this representation.

We begin by mapping *X* into a representation *Z*∈ℝ^*k*^ using an encoder function $f_{\phi }:{\mathbb{R}}^{d}\to {\mathbb{R}}^{k}$, parameterized by ϕ. That is, *Z* = *f*_ϕ_(*X*). Rather than directly using *Z* to predict the outcome, we introduce a hypernetwork mechanism. A hypernetwork is a neural network that outputs the parameters of another network. Specifically, a function $g_{\psi }:{\mathbb{R}}^{k}\to {\mathbb{R}}^{m}$, parameterized by ψ maps the representation *Z* to a weight vector ω∈ℝ^*m*^, which defines the parameters of a second, fixed-architecture neural network *h*_ω_:ℝ → ℝ. This target network takes the treatment variable *T* as input and its output yields the predicted outcome Ŷ:


$$\begin{array}{l} \omega =g_{\psi }\left(Z\right),\;Ŷ=h_{\omega }\left(T\right). \end{array}$$

Crucially, while the functional form (i.e., architecture) of *h*_ω_ is fixed and non-trainable *post hoc*, its weights ω are dynamically generated for each input instance via the hypernetwork. The overall model thus defines a composite function:


$$\begin{array}{l} Ŷ=h_{g_{\psi }\left(f_{\phi }\left(X\right)\right)}\left(T\right). \end{array}$$

The model is trained end-to-end by minimizing a prediction loss, such as the mean squared error between the observed outcomes and the predicted outcomes:


$$\begin{array}{l} ℒ\left(\phi ,\psi \right)=E_{\left(X,T,Y\right)}\left[{\left(Y-h_{g_{\psi }\left(f_{\phi }\left(X\right)\right)}\left(T\right)\right)}^{2}\right]. \end{array}$$

**Figure 3:**
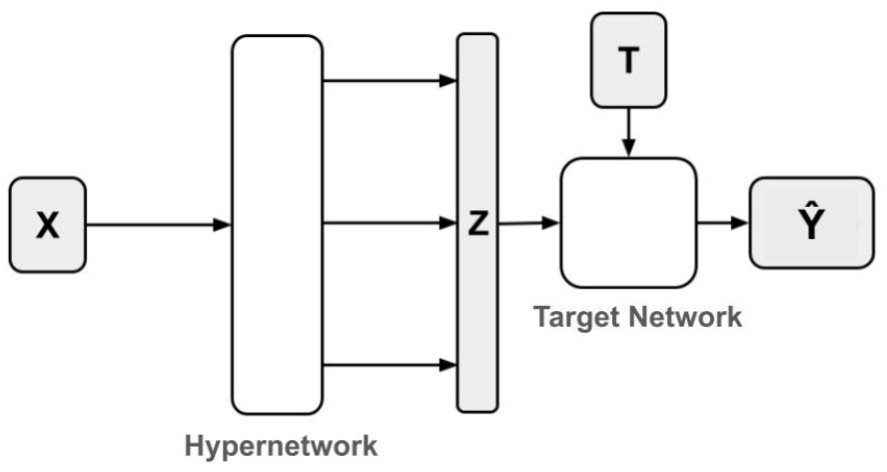
Illustration of HNet Architecture. A shared representation layer *Z* is used to compute the weights of instance-level fixed networks that model θ_*X*_(*T*).

This architecture induces a structured form of adaptation: the representation *Z* encodes individual-specific information, which is then used to instantiate a custom predictor *h*_ω_ for each individual. The hypernetwork thus acts as a meta-model that generates individualized outcome models conditioned on latent features, thereby enabling a flexible and context-dependent estimation of potential outcomes. A schematic overview of the proposed model architecture is provided in Figure 3.

#### 2.3.2 Higher dimensional treatments

While the model above is described for the case of a continuous univariate treatment *T*∈ℝ, the architecture naturally extends to higher-dimensional treatments *T*∈ℝ^*p*^. In this case, the target network *h*_ω_ simply takes a vector-valued input and outputs a scalar prediction, i.e., *h*_ω_ : ℝ^*p*^ → ℝ. The hypernetwork continues to generate the weights ω conditioned on the representation Z, and no changes are required to the overall training objective or meta-model structure. As such, the framework maintains its flexibility and personalization capabilities regardless of the dimensionality of the treatment space.

#### 2.3.3 Additional causal regularization

While the current training objective relies on a standard mean squared error prediction loss to isolate the architectural benefits of the hypernetwork parameterization without conflating them with regularization choices, the modularity of HNet allows for the seamless integration of causal-inference-specific regularization techniques. To address settings with strong confounding several balancing strategies can be incorporated into the objective function.

First, Inverse Probability Weighting (IPW) or density-ratio weighting can be integrated by modeling the continuous propensity score, *p*(*t*|*x*), via a conditional density estimator. This approach directly reweights the base prediction loss, yielding a doubly robust objective without requiring architectural changes.

Second, distribution matching can be applied within the shared representation space, *Z*. By enforcing Integral Probability Metrics on the outputs of the encoder network, the model is encouraged to learn treatment-invariant representations, extending principles established by architectures like TARNet to the continuous domain.

Finally, drawing on the structural regularizations seen in Dragonnet and VCNet, a targeted regularization term can be added to the loss function. This specifically reduces bias in the average dose-response estimation, serving as a complement to the highly individualized instance-level predictions generated by the hypernetwork.

#### 2.3.4 Theoretical distinctions and inductive biases

A hypernetwork-based parameterization allows the counterfactual function to vary arbitrarily across the covariate space, leveraging the universal approximation properties of hypernetworks. In contrast, B-spline approaches like VCNet constrain the treatment-response curve to a fixed function space. Ultimately, these models encode distinct inductive biases: the unconstrained flexibility of hypernetworks excels in capturing complex, heterogeneous effects, while the structured regularization of splines may be preferable in other specific scenarios.

#### 2.3.5 Limitations

While our method offers a flexible and powerful framework for estimating treatment effects, it operates within the broader context of continuous treatment effect estimation, which presents its own inherent challenges. One such challenge is the need for a strong overlap condition, typically formalized as requiring the propensity score to satisfy *g*(*t*∣*x*)≥*c*>0 for all treatment values *T* and covariates *X*. In real-world applications with near-deterministic treatment assignments, this assumption may be violated, leading to small or vanishing propensity scores that can destabilize estimation for any model.

Beyond this structural limitation of the problem, our method itself also has practical constraints. It can be sensitive to the size of the available dataset—particularly in settings where the treatment-confounder relationship is complex and high-capacity models like neural networks are used. In such cases, limited data may lead to overfitting or insufficient representation of the underlying causal structure. Additionally, the method's performance depends on the careful tuning of multiple hyperparameters, including network architecture, learning rate, regularization strength, and batch size. This tuning process can be computationally intensive and may pose a barrier in applications where computational resources or validation data are limited.

Additionally, while our method provides a flexible and effective framework for estimating treatment effects, it does not explicitly address several known sources of bias that have been the focus of other approaches. In particular, issues such as continuous treatment imbalance (Wang et al., 2020), spurious interactions (Pros and Vitrià, 2025), and biases introduced by the mismatch between the observed data distribution and the independence needed for counterfactual estimation (Nagalapatti et al., 2024) remain unmitigated in our current formulation. These limitations suggest that the method may benefit from being integrated with complementary techniques specifically designed to address these challenges. Future work could explore such hybrid approaches to enhance robustness and broaden the applicability of the framework in more complex or biased settings.

## 3 Experiments

In this section, we evaluate the performance of our proposed model using widely adopted treatment effect estimation benchmarks. Since counterfactual outcomes are inherently unobservable in real-world settings, direct evaluation is not feasible. To overcome this challenge, we employ standard experimental datasets commonly used in the causal inference literature (Yao et al., 2021). For binary treatment effect estimation, we use the IHDP dataset (Hill, 2011) and results can be found in Appendix D. Given our primary focus on continuous treatment effect estimation, we further evaluate our approach on three appropriate datasets: a fully synthetic dataset introduced in Nie et al. (2021), and two semi-synthetic datasets, Continuous IHDP (Hill, 2011; Nie et al., 2021) and TCGA (Weinstein et al., 2013). We benchmark our model against several baseline models using established evaluation metrics for treatment effect estimation.

### 3.1 Experimental setup

All the experiments are run on a MacBook Pro (Apple Inc., Cupertino, CA, USA) with an Apple M2 Max chip and 32GB memory. All the code for reproducing the experiments can be found in: https://anonymous.4open.science/r/Instance_Specific_Continuous_Treatments-C045/. Details regarding hyperparameter tuning and evaluation metrics are provided in Appendices B, C, as well as in the accompanying code. In terms of computational complexity, HNet remains in line with other neural network-based estimators. Both training and inference times are in the order of seconds for the datasets evaluated in this study, taking approximately twice the computational time of a standard Multi-Layer Perceptron (MLP).

In this work, we assume all our datasets are originally composed of valid adjustment sets, i.e. the backdoor criterion can be satisfied and assumptions 1 and 2 hold. In addition to HNet, we evaluate the benchmarks using neural network-based S-Learner models. The SLearn NN baseline serves as a direct ablation study for our proposed architecture; it effectively replaces the hypernetwork mechanism with a standard Multi-Layer Perceptron (MLP) that takes the concatenated representation and treatment variables as input, allowing us to isolate the specific value added by the hypernetwork. For specialized continuous treatment effect architectures, we restricted our baselines to models and datasets for which the original authors provided verified hyperparameter configurations. Consequently, VCNet is evaluated on the synthetic and Continuous IHDP datasets, while models such as SCIGAN and DRNet are exclusively compared on the TCGA datasets, mirroring their original experimental setups. This strict inclusion criterion ensures a fair and rigorous comparison, preventing the misrepresentation of baseline models that could arise from suboptimal, *ad-hoc* hyperparameter tuning on novel datasets.

### 3.2 Continuous treatment effects estimations

#### 3.2.1 Synthetic dataset

We evaluate the model in the synthetic dataset introduced in Nie et al. (2021). The dataset consists of 100 instances of 500 training samples and 200 test samples. Details of the data generation process are provided in Appendix A.

In Table 1, we present the performance of various models on the synthetic dataset introduced by Nie et al. (2021). With a MISE error of 0.073 and AMSE error 0.015, HNet outperforms all baselines, achieving the lowest error in both metrics, suggesting highly accurate estimation of the individual treatment effect function.

**Table 1:** Performance of different models on the synthetic dataset from Nie et al. (2021).

Model	MISE	AMSE
SLearn NN	0.375 ± 0.011	0.145 ± 0.008
VCNet	0.150 ± 0.002	0.016 ± 0.001
HNet	0.073 ± 0.003	0.015 ± 0.001

We report Mean Integrated Squared Error (MISE) and Average Mean Squared Error (AMSE). Lower values indicate better performance.

VCNet performs competitively, with an MISE and AMSE reflecting solid performance consistent with its design for continuous treatment settings. However, it is still outperformed by HNet by an important margin in the MISE metric of this benchmark. For the AMSE metric, both VCNet and HNet perform similarly.

For the S-learner baseline, SLearn NN records the highest errors across both metrics (0.375 for MISE and 0.145 for AMSE). These results suggest that simple neural network-based S-learners may struggle in this synthetic setting.

Overall, HNet demonstrates strong generalization and estimation capabilities, highlighting its effectiveness in capturing treatment heterogeneity in synthetic scenarios with continuous treatments.

#### 3.2.2 Continuous IHDP

This dataset is a version of the IHDP dataset adapted for continuous treatment effect estimation. It uses the same covariates as the original dataset, and, following Nie et al. (2021), treatment and response are generated to allow comparison on continuous treatments, details on the generation can be found in Appendix A. For consistency and comparability, we adopt the same hyperparameter tuning strategy described in Nie et al. (2021).

The results in Table 2 show the performance of several models on the continuous treatment version of the IHDP dataset, evaluated using MISE and AMSE. The S-Learner with a neural network base (Slearn NN) performs poorly on both metrics, suggesting that this approach may suffer from high variance or inadequate model capacity when not explicitly tailored to the continuous treatment setting.

**Table 2:** Performance comparison of models on the continuous IHDP dataset using two evaluation metrics: Mean Integrated Squared Error (MISE) and Average Mean Squared Error (AMSE).

Model	MISE	AMSE
SLearn NN	6.130 ± 0.067	3.699 ± 0.063
VCNet	1.573 ± 0.050	0.460 ± 0.036
HNet	1.352 ± 0.053	0.236 ± 0.035

Lower values indicate better estimation of the continuous dose-response function.

Among the models specifically designed for continuous treatments, HNet outperforms VCNet on both MISE and AMSE, achieving values of 1.352 and 0.236, respectively, compared to VCNet's 1.573 and 0.460. These results suggest that explicitly accounting for treatment relevance is important in this setting, and that modeling treatment effects as a hypernetwork leads to improved performance.

#### 3.2.3 TCGA

The TCGA dataset (Weinstein et al., 2013), obtained from The Cancer Genome Atlas project, consists of data on various types of cancer in 9,659 individuals. Each individual is characterized by 4000 dimensions of gene expression covariates. These covariates are log-normalized and further normalized to have unit variance. The treatment variable represents the dosage of the drug taken by the patient, while the synthetic response models the risk of cancer recurrence. In our experiments, we use three versions of the TCGA dataset proposed and detailed in Schwab et al. (2020); Bica et al. (2020) referred to as TCGA(0), TCGA(1), and TCGA(2), running each 10 times. To maintain consistency with the original literature proposing these benchmarks and to facilitate direct comparison with previously reported baselines, we report the results for this dataset using the $\sqrt{\text{MISE}}$ metric.

In Table 3, we observe the model performances across the three TCGA dataset variants. The HNet model consistently outperforms SLearn Neural Network (NN) across all datasets, with substantially lower MISE and AMSE values. SLearn NN, on the other hand, demonstrates unstable behavior with much higher errors, indicating poor suitability for this task.

**Table 3:** Model performance on the TCGA(0), TCGA(1), and TCGA(2) datasets.

Dataset	Model	$\sqrt{MISE}$	AMSE
TCGA (0)	SLearn NN	2.582 ± 0.002	6.691 ± 0.007
	HNet	0.275 ± 0.013	0.042 ± 0.006
TCGA (1)	SLearn NN	3.513 ± 0.009	5.036 ± 0.010
	HNet	0.204 ± 0.008	0.040 ± 0.003
TCGA (2)	SLearn NN	7.664 ± 0.025	18.431 ± 0.086
	HNet	0.616 ± 0.067	0.916 ± 0.044

MISE (lower is better) measures error in estimating the response curve, while AMSE evaluates treatment effect estimation accuracy.

Table 4 summarizes the combined performance across all datasets. The HNet model achieves the lowest MISE (0.411), indicating superior accuracy in estimating response functions. These findings suggest that HNet is competitive and performs well in treatment effect estimation. In contrast, SLearn NN performs substantially worse on both metrics, indicating limited reliability in this context.

**Table 4:** Combined model performance across all TCGA datasets.

Model	$\sqrt{MISE}$	AMSE
SCIGAN (Bica et al., 2020)	1.890 ± 0.050	–
DRNet (Schwab et al., 2020)	3.640 ± 0.120	–
DRN-W (Schwab et al., 2020)	3.710 ± 0.120	–
GPS (Imbens, 2000)	4.830 ± 0.010	–
SLearn NN	5.091 ± 0.013	12.745 ± 0.048
HNet	0.411 ± 0.034	0.524 ± 0.024

This final evaluation aggregates results to highlight overall model reliability.

## 4 Discussion and conclusion

In this work, we presented a novel neural network-based approach for estimating continuous treatment effects by leveraging hypernetworks to model counterfactual outcomes across treatment levels. Our method extends established principles of binary treatment effect estimation to the continuous setting, addressing the key challenge of treatment relevance. By generating weights for a fixed network that predicts potential outcomes, our architecture preserves the causal significance of the treatment variable while maintaining the flexibility of deep learning models.

Extensive experiments on synthetic and semi-synthetic datasets demonstrated that our approach consistently outperforms existing methods in terms of precision. These results underscore the benefits of explicitly modeling the relationship between treatment levels and outcomes, particularly in scenarios involving high-dimensional confounders or non-linear treatment-response dynamics.

Our contribution advances the field of causal machine learning by bridging the gap between binary and continuous treatment effect estimation. By combining the expressive capacity of neural networks with principled causal assumptions, we offer a flexible and interpretable framework for inferring causal relationships from observational data.

Future research directions include exploring robustness to unmeasured confounding through sensitivity analysis and integrating validated bias mitigation methods such as adversarial training to address continuous treatment imbalance or the prevention of spurious interactions.

## Data Availability

The datasets presented in this study can be found in online repositories. The names of the repository/repositories and accession number(s) can be found in the article/Supplementary material.

## Footnotes

1 The *do*-notation denotes an *intervention* in a causal model rather than a passive observation. Writing do(*T* = *t*) represents actively setting the treatment variable *T* to *t* and considering the resulting distribution of outcomes. See Pearl (2009).
