# Supplementary material for: Learning instance-specific counterfactual models for continuous treatments using hypernetworks
Source: Front Artif Intell. 2026 May 8;9:1819009. doi: 10.3389/frai.2026.1819009 (PMC13194395; doi:10.3389/frai.2026.1819009)
Supplement: Supplementary file 1 [file Table_1.pdf]

frontiers

Learning Instance-Specific Counterfactual Models for Continuous Treatments using Hypernetworks

Roger Pros [1,*], **Jordi Vitrià** [1]

[1] *Departament de Matemàtica i Informàtica, Universitat de Barcelona, Barcelona, 08007, Spain*

Correspondence*:
Roger Pros
roger.pros@ub.edu

APPENDIX A - DATA GENERATION DETAILS

Synthetic dataset (Nie et al., 2021)

The data is generated as follows: $x_j \overset{\text{i.i.d.}}{\sim} \text{Unif}[0,1]$, where x_j is the j-th dimension of $x \in \mathbb{R}^6$, and

$$e_t \mid x = \frac{10\sin(\max(x_1,x_2,x_3)) + \max(x_3,x_4,x_5)^3}{(1+(x_1+x_5)^2)} + \sin(0.5x_3)\left(1+\exp(x_4-0.5x_3)\right) + x_3^2 + 2\sin(x_4) + 2x_5 - 6.5$$ [illegible]

$$y \mid x,t = \cos\left(2\pi(t-0.5)\right)\left(t^2 + \frac{4\max(x_1,x_6)^3}{(1+2x_3^2)}\sin(x_4)\right) + \mathcal{N}(0,0.25),$$

where $t = (1+\exp(-\tilde{t}))^{-1}$.

Continuous IHDP (Nie et al., 2021)

Treatment and response are generated using:

$$\begin{aligned}
\tilde{t} \mid \mathbf{x} &= \frac{2x_1}{(1+x_2)} + \frac{2\max(x_3,x_5,x_6)}{0.2+\min(x_3,x_5,x_6)} \\
&\quad + 2\tanh\left(5\frac{\sum_{i\in S_{\text{dis},2}}(x_i-c_2)}{|S_{\text{dis},2}|}\right) - 4 + \mathcal{N}(0,0.25), \\
y \mid \mathbf{x}, t &= \frac{\sin(3\pi t)}{1.2-t}\left(\tanh\left(5\frac{\sum_{i\in S_{\text{dis},1}}(x_i-c_1)}{|S_{\text{dis},1}|}\right)\right. \\
&\quad \left. + \frac{\exp(0.2(x_1-x_6))}{0.5+5\min(x_2,x_3,x_5)}\right) + \mathcal{N}(0,0.25),
\end{aligned}$$

where $t = (1 + \exp(-\tilde{t}))^{-1}$, $S_{\text{con}} = \{1, 2, 3, 5, 6\}$ is the index set of continuous features, $S_{\text{dis},1} = \{4, 7, 8, 9, 10, 11, 12, 13, 14, 15\}$, $S_{\text{dis},2} = \{16, 17, 18, 19, 20, 21, 22, 23, 24, 25\}$ and $S_{\text{dis},1} \cup S_{\text{dis},2} = [25] - S_{\text{con}}$. Here $c_1 = \mathbb{E}\left[\frac{\sum_{i \in S_{\text{dis},1}} x_i}{|S_{\text{dis},1}|}\right]$, $c_2 = \mathbb{E}\left[\frac{\sum_{i \in S_{\text{dis},2}} x_i}{|S_{\text{dis},2}|}\right]$.

APPENDIX B - EVALUATION METRICS

Binary evaluation metrics

For binary treatment effect estimation, two common evaluation metrics are:

- **Precision in Estimation of Heterogeneous Effect (PEHE)**: Measures the mean squared error between the estimated individual treatment effects and the true individual treatment effects.

$$\sqrt{PEHE} = \sqrt{\frac{1}{n}\sum_{i=1}^{n}(\hat{\tau}_i - \tau_i)^2}$$

  where $\hat{\tau}_i = \hat{Y}_i(1) - \hat{Y}_i(0)$ and $\tau_i = Y_i(1) - Y_i(0)$.

- **Average Treatment Effect Error (ATE)**: Measures the difference in average potential outcomes under treatment and control.

$$\epsilon_{ATE} = \left|\frac{1}{n}\sum_{i=1}^{n}\left(\hat{Y}_i(1) - \hat{Y}_i(0)\right) - \frac{1}{n}\sum_{i=1}^{n}(Y_i(1) - Y_i(0))\right|$$

Continuous evaluation metrics

For continuous treatment effect estimation, the following metrics are used:

- **Mean Integrated Squared Error (MISE)**: Measures the average integrated squared error across individuals.

$$\text{MISE} = \frac{1}{n}\sum_{i=1}^{n}\left[\int_{\mathcal{T}}(\hat{y}_i(t) - y_i(t))^2\, dt\right]$$

- **Average Mean Squared Error (AMSE)**: Measures the integrated squared error of the average response curve, weighted by the treatment distribution $p(t)$.

$$\text{AMSE} = \int_{\mathcal{T}}\left[\frac{1}{n}\sum_{i=1}^{n}(\hat{y}_i(t) - y_i(t))\right]^2 p(t)dt$$

  We approximate the AMSE by applying all the t values present in the current dataset to each individual, yielding

$$\widehat{\text{AMSE}} = \frac{1}{|\mathcal{T}|}\sum_{t \in \mathcal{T}}\left[\frac{1}{n}\sum_{i=1}^{n}(\hat{y}_i(t) - y_i(t))\right]^2,$$

  where $|\mathcal{T}|$ is the number of different treatment values.

Relationsip between MISE and AMSE

The key distinction between MISE and AMSE lies in the order of operations. MISE computes the squared error for each individual before averaging across the population, thereby penalizing inaccuracy at the individual level. AMSE, by contrast, first averages errors across individuals at each treatment value and then squares the result, thus capturing only the deviation of the estimated population-mean dose–response curve from its true counterpart. Consequently, AMSE is the appropriate metric when the estimand of interest is a population-level quantity such as the average dose–response function, whereas MISE provides a stricter assessment of estimation quality when individual-level accuracy is required, as in personalized treatment recommendations.

APPENDIX C - HYPERPARAMETERS

Target network size sensitivity

The size and capacity of the target network is a highly sensitive hyperparameter that requires careful fine-tuning. During our experiments, we observed that suboptimal configurations of the target network size could cause the model's overall performance to drop by up to 80%. To rigorously navigate this sensitivity and ensure that our results reflect the true architectural benefits, we utilized Bayesian optimization through the Hyperopt (Bergstra et al., 2013) package to systematically fine-tune these parameters across all models.

Model hyperparameters

SLearn NN:

- **[Hidden Layers]** The number of hidden layers in the neural network, selected from the set {3, 4, 5}.
- **[Neurons per Layer]** The number of neurons in each hidden layer, chosen from {20, 35, 50, 65, 80}.
- **[Learning Rate]** The learning rate for the optimizer, sampled from {0.05, 0.005, 0.001, 0.0005, 0.0001}.
- **[Dropout]** Dropout rate, selected from the range {0, 0.5}.

VCNet: Used same hyperparameters as in the original work.

HNet:

- **[Hidden Layers]** The number of hidden layers in the neural network, selected from the set {3, 4, 5}.
- **[Neurons per Layer]** The number of neurons in each hidden layer, chosen from {20, 35, 50, 65, 80}.
- **[Learning Rate]** The learning rate for the optimizer, sampled from {0.05, 0.005, 0.001, 0.0005, 0.0001}.
- **[Dropout]** Dropout rate, selected from the range {0, 0.5}.
- **[Output Dimension]** The dimensionality of the output layer, selected from even integers in the range {12, 100}.
- **[Hidden Network Layers]** The number of layers in the hidden network, chosen from {0, 1, 2, 3}.
- **[Hidden Network Activation]** The activation function for the hidden layers, selected from {ELU, ReLU, Linear}.

- **[Spectral Normalization]** A boolean flag indicating whether spectral normalization is applied, chosen from {True, False}.
- **[Negative Gaussian]** A boolean flag for applying negative Gaussian regularization, chosen from {True, False}.

APPENDIX D - BINARY TREATMENT EFFECTS ESTIMATION - IHDP

MODEL	$\sqrt{PEHE}$	ϵ_{ATE}
BNN (Johansson et al., 2016)	2.10 ± .10	0.42 ± .03
CFRW (Shalit et al., 2017)	0.76 ± .00	0.27 ± .01
CEVAEs (Louizos et al., 2017)	2.60 ± .10	0.46 ± .02
GANITE (Yoon et al., 2018)	2.40 ± .40	0.49 ± .05
TARNet (Shalit et al., 2017)	1.28 ± .05	0.20 ± .01
Dragonnet (Shi et al., 2019)	1.29 ± .06	0.20 ± .01
BCAUSS (Tesei et al., 2023)	0.96 ± .03	0.15 ± .01
BCAUSS + SIP (Pros and Vitrià, 2025)	0.75 ± .03	0.13 ± .01
HNet	1.14 ± 0.04	0.22 ± 0.01

Table 1. Performance of various causal inference models on the IHDP dataset. We report $\sqrt{PEHE}$ (the root of the Precision in Estimation of Heterogeneous Effects error) and ϵ_{ATE} (the Absolute Error in Average Treatment Effect). Lower values indicate better performance.

We evaluate the model in the popular benchmark IHDP. The Infant Health and Development Program (IHDP) is a study that used randomization to examine the effects of home visits by specialist doctors on the cognitive test scores of premature infants. The dataset[1] was first used by (Hill, 2011) to evaluate algorithms for estimating treatment effects. To create an observational dataset, non-random subsets of treated individuals were removed, resulting in selection bias. The outcomes in the dataset were generated using the original covariates and treatments. The dataset consists of 747 subjects and 25 variables. Following the recent literature, we used the simulated outcome implemented in the NPCI package (Dorie, 2016), which is composed of 1000 repetitions of the experiment. We averaged our results over 1000 train/validation/test splits with ratios 70/20/10.

In Table 1, we report the performance of several models on the IHDP benchmark dataset. These results demonstrate that BCAUSS + SIP achieves the lowest error in both $\sqrt{PEHE}$ and ϵ_{ATE}, indicating superior estimation accuracy under the presence of treatment selection bias and violations of the positivity assumption, known challenges in this dataset.

To assess the generality of our continuous treatment architecture, we additionally evaluated HNet on the binary IHDP benchmark. As expected, HNet does not achieve parity with state-of-the-art methods specifically tailored for binary treatments and selection bias correction, such as BCAUSS + SIP, which achieves a $\sqrt{PEHE}$ of 0.75 ± 0.03. Because HNet lacks separate treatment/control heads and binary-specific regularization, this performance gap is entirely anticipated. However, with a $\sqrt{PEHE}$ of 1.14 ± 0.04 and an ϵ_{ATE} of 0.22 ± 0.01 , HNet performs reasonably well out-of-the-box, placing alongside established foundational baselines like TARNet and Dragonnet. Rather than claiming competitive parity in this domain, these findings serve as a generality check, confirming that the hypernetwork parameterization remains viable and robust when applied to discrete settings, even without binary-specific inductive biases.

[1] The dataset can be accessed at `www.fredjo.com`.

REFERENCES

Bergstra, J., Yamins, D., and Cox, D. (2013). Making a science of model search: Hyperparameter optimization in hundreds of dimensions for vision architectures. In *International conference on machine learning* (PMLR), 115–123

Dorie, V. (2016). Npci: Non-parametrics for causal inference. *URL: https://github. com/vdorie/npci* 11, 23

Hill, J. L. (2011). Bayesian nonparametric modeling for causal inference. *Journal of Computational and Graphical Statistics* 20, 217–240

Johansson, F., Shalit, U., and Sontag, D. (2016). Learning representations for counterfactual inference. In *International conference on machine learning* (PMLR), 3020–3029

Louizos, C., Shalit, U., Mooij, J. M., Sontag, D., Zemel, R., and Welling, M. (2017). Causal effect inference with deep latent-variable models. *Advances in neural information processing systems* 30

Nie, L., Ye, M., Liu, Q., and Nicolae, D. (2021). Vcnet and functional targeted regularization for learning causal effects of continuous treatments. *arXiv preprint arXiv:2103.07861*

Pros, R. and Vitrià, J. (2025). Preventing spurious interactions: A new inductive bias for accurate treatment effect estimation. *IEEE Access*

Shalit, U., Johansson, F. D., and Sontag, D. (2017). Estimating individual treatment effect: generalization bounds and algorithms. In *International conference on machine learning* (PMLR), 3076–3085

Shi, C., Blei, D., and Veitch, V. (2019). Adapting neural networks for the estimation of treatment effects. *Advances in neural information processing systems* 32

Tesei, G., Giampanis, S., Shi, J., and Norgeot, B. (2023). Learning end-to-end patient representations through self-supervised covariate balancing for causal treatment effect estimation. *Journal of Biomedical Informatics* 140, 104339

Yoon, J., Jordon, J., and Van Der Schaar, M. (2018). Ganite: Estimation of individualized treatment effects using generative adversarial nets. In *International conference on learning representations*
